# Serial testing for latent tuberculosis using QuantiFERON-TB Gold In-Tube: A Markov model

**DOI:** 10.1038/srep30781

**Published:** 2016-07-29

**Authors:** Mark W. Moses, Alice Zwerling, Adithya Cattamanchi, Claudia M. Denkinger, Niaz Banaei, Sandra V. Kik, John Metcalfe, Madhukar Pai, David Dowdy

**Affiliations:** 1 Department of Epidemiology, Johns Hopkins Bloomberg School of Public Health, Baltimore, USA; 2 Department of Medicine, UCSF, San Francisco, USA; 3 Tuberculosis and Hepatitis Programme, FIND, Geneva, Switzerland; 4 Department of Medicine, Stanford University, Palo Alto, USA; 5 Evidence Team, KNCV, The Hague, Netherlands; 6 Department of Epidemiology & Biostatistics and McGill International TB Centre, McGill University, Montreal, Canada

## Abstract

Healthcare workers (HCWs) in low-incidence settings are often serially tested for latent TB infection (LTBI) with the QuantiFERON-TB Gold In-Tube (QFT) assay, which exhibits frequent conversions and reversions. The clinical impact of such variability on serial testing remains unknown. We used a microsimulation Markov model that accounts for major sources of variability to project diagnostic outcomes in a simulated North American HCW cohort. Serial testing using a single QFT with the recommended conversion cutoff (IFN-g > 0.35 IU/mL) resulted in 24.6% (95% uncertainty range, UR: 23.8–25.5) of the entire population testing false-positive over ten years. Raising the cutoff to >1.0 IU/mL or confirming initial positive results with a (presumed independent) second test reduced this false-positive percentage to 2.3% (95%UR: 2.0–2.6%) or 4.1% (95%UR: 3.7–4.5%), but also reduced the proportion of true incident infections detected within the first year of infection from 76.5% (95%UR: 66.3–84.6%) to 54.8% (95%UR: 44.6–64.5%) or 61.5% (95%UR: 51.6–70.9%), respectively. Serial QFT testing of HCWs in North America may result in tremendous over-diagnosis and over-treatment of LTBI, with nearly thirty false-positives for every true infection diagnosed. Using higher cutoffs for conversion or confirmatory tests (for initial positives) can mitigate these effects, but will also diagnose fewer true infections.

Health care workers (HCWs) are at risk for exposure to tuberculosis (TB) and are routinely tested for latent TB infection (LTBI) as part of most infection control programs in North American hospitals^1^,^2^,^3^. Traditionally, HCWs have been screened with the Tuberculin Skin Test (TST), and preventive therapy provided for those testing positive^1^,^4^, but over the last decade, interferon-gamma release assays (IGRAs) have been introduced as an alternative to the TST^5^,^6^,^7^. IGRAs–including the QuantiFERON-TB Gold In-Tube (QFT, Qiagen, Valencia, USA) and T-SPOT.TB (Oxford Immunotec, Oxfordshire, UK)–offer several advantages over the TST, including improved specificity (particularly in BCG vaccinated populations)^7^, *in vitro* testing, and the requirement that patients make only a single visit. As a result, many hospitals in the United States have begun to use IGRAs for serial testing of HCWs; this practice has revealed unexpectedly frequent conversions and reversions among IGRA results^8^,^9^,^10^,^11^,^12^,^13^, the clinical implications of which remain unclear.

IGRA results are known to have multiple sources of variability (unrelated to TB infection), which may contribute to the observed variation in test results^14^. The clinical implications of such variability in the setting of serial testing remain unknown. As a result, it is uncertain how best to implement IGRAs for serial testing of HCWs–for example, to reduce the occurrence of false positives among individuals subjected to numerous repeat tests, serial testing might warrant a different cutoff for a conversion or confirmatory testing of initial positive results. A recent systematic review^15^ summarized the evidence on potential sources of IGRA variability; evidence was strongest for boosting of IGRA (and specifically, QFT) responses by previously placed TSTs, delays in sample processing prior to incubation, and variations in the blood volume drawn. The evidence base for quantifying variability in serial testing by T-SPOT.TB (which has fundamental methodological differences in performance, compared to QFT) was somewhat weaker, and sources of T-SPOT.TB variability are likely to be different from those related to QFT. We therefore constructed a simulation of hypothetical North American HCWs undergoing serial testing with QFT, taking into account best estimates of both within-subject variability (probabilities of conversion and reversion in populations at very low risk of TB infection) and two important additional sources of variability (incubation delays and variation in blood volume). We use this simulated population to project the outcomes of serial QFT testing over a period of ten years.

## Methods

### Overview

**Figure 1:**
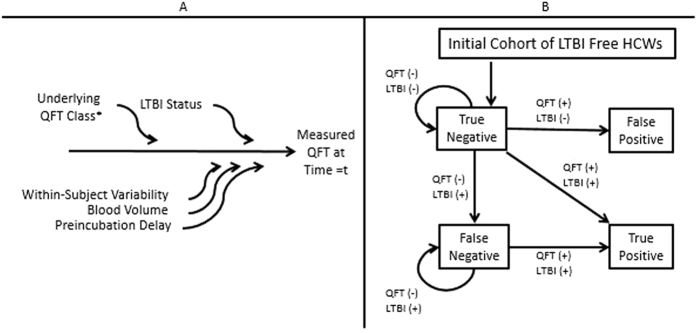
Markov model of serial QuantiFERON-TB (QFT) testing. Panel A illustrates the method by which each individual’s measured QFT result was calculated at each year *t*. This result reflects the individual’s initial underlying QFT “class”, within-subject variability from year to year, and two additional sources of variability, as described in the methods. *Within-subject variability is incorporated through annual random draws from distributions based on each individual’s initial underlying QFT class. Panel B shows the four-state Markov model. We assume that healthcare workers (HCWs) enter as true negatives and cycle between other states on an annual basis for 10 years based on yearly QFT result (according to different conversion cutoffs and testing strategies, as described in the Methods) and true latent tuberculosis infection (LTBI) status. QFT: Quantiferon-TB Gold IN-Tube test, LTBI: latent tuberculosis infection, TST: tuberculin skin test, HCW: health care workers.

We used a microsimulation Markov model to project diagnostic outcomes in a simulated HCW cohort under different serial screening strategies. The simulated cohort comprised 10,000 hypothetical HCWs at a representative North American institution undergoing serial LTBI screening using QFT every year over a period of ten years. The model incorporates the major sources of variability in serial QFT results as well as different serial screening strategies, including different cutoffs for a QFT conversion and confirmatory testing with different degrees of correlation (modeled as Pearson correlation coefficients) between initial and confirmatory results (Fig. 1).

### Modeling Approach

To accomplish the model objectives, the model first assigns each individual HCW to one of three “QFT classes” based on the IFN-gamma response (0.00–0.19 International Units (IU)/mL [“class 1”], 0.20–0.99 IU/mL [“class 2”], or ≥1.00 IU/mL [“class 3”]), reflecting the distribution of initial QFT results from a large, multi-center study of HCWs at low risk of TB infection who were tested serially under semi-controlled conditions^8^. This “class” then dictates the HCW’s future “underlying” QFT results on a yearly basis under random within-subject variability alone, as follows. First, we take the initial QFT results from the aforementioned study, dividing these QFT results into the same three classes. Thus, for example, individuals in this study whose initial QFT result was 0.00–0.19 IU/mL would be assigned to “class 1”. Then, we fit a gamma distribution to the repeat QFT results in each class, again using the serial testing data from the aforementioned study. (For example, if nine of ten patients whose initial QFT results were <0.20 IU/mL in the source study had a repeat value of 0.00 IU/mL, and one had a repeat value of 0.40 IU/mL, we would fit a gamma distribution to those values, to apply to the simulated HCWs in class 1.) Gamma distributions were selected because their lower bound is zero, but results are distributed in a right-skewed fashion with an infinite upper bound. The cutoffs for the three classes were selected based on natural cut-points in the data^8^, and to be maximally conservative (i.e., reducing the probability of false conversions on serial testing). Finally, to account for within-subject variability within each class, we assign a different “underlying” QFT value to each simulated HCW on a yearly basis, each yearly value representing a random draw from the distribution corresponding to that HCW’s class. We assume for simplicity that, at intake, all members of the cohort are free of actual TB infection.

In subsequent years, in addition to the within-subject variability as described above, an annual risk of true TB infection is applied. In the baseline scenario, we assume a high-risk HCW population in a North American healthcare institution in which 0.1% of the cohort acquires LTBI each year^16^,^17^. This annual risk of infection is varied in scenario analyses. The process of true infection is modeled by replacing the previous “underlying” QFT result with a new “underlying” value selected by randomly sampling from the distribution of positive QFT test results in the same study (i.e., assuming that the distribution of positive results in this study is similar to the distribution of true-positive results following true TB infection) [8].

After assigning an “underlying” QFT value to each simulated HCW in each year (to account for within-subject variability and the possibility of true infection)^8^,^10^,^18^, the model incorporates additional variation in QFT results according to two additional sources of variability (Fig. 1A): (1) variation in blood volume: 1 mL+/− 0.2 mL^19^ (assuming variation in either direction on 5% of all blood draws, for a total of 10%); and (2) pre-analytical delay of six or 12 hours^20^,^21^ (assuming each occurs in 5% of blood draws, for a total of 10%). We performed a sensitivity analysis that also included variability from TST boosting^22^,^23^,^24^, assuming boosting in 2% of all blood draws. We considered TST boosting an unlikely source of variability in individuals enrolled in a QFT screening program and thus excluded it from all other analyses. Each of these sources of variability is modeled as a beta distribution (with defined upper and lower bounds); values and ranges for change in QFT results are presented in Table 1. For HCWs randomly selected to experience each source of variability, a value would be selected from the corresponding distribution and added (or subtracted) from the “underlying” QFT value to obtain the final measured QFT value in that year. Variability from blood volume, pre-analytical delay, and TST boosting is assumed to occur independently between each other and over time, such that the occurrence of one of these sources of variability in one year has no bearing on whether it or another source of variability occurs again in the current year or in a subsequent year.

The model can thus be represented mathematically as follows. Given an “underlying” QFT value (*a*_*st*_) in each year, additional variability in QFT results is added according to Equation 1:


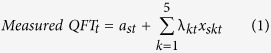


where *a*_*st*_ is the “underlying” QFT value (conditional on TB infection status) as described above*, k* represents the additional sources of variability for which sufficient data were available in the systematic review (pre-incubation delay, blood volume, plus true TB infection^15^), λ_*kt*_ is an indicator variable drawn from a binomial distribution corresponding to the probability that *k* occurred in year *t* (1 = yes, 0 = no), and *x*_*skt*_ is a value randomly drawn from a beta distribution fit to the measured variability in QFT results among people whose initial QFT value was in class *s*, and whose repeat tests were known to have been exposed to each item *k*, using data from the systematic review^15^.

After incorporating these sources of variability and true LTBI infection, measured QFT results are truncated between 0 and 10 IU/mL (corresponding to manufacturer’s guidelines), and a test result (positive or negative) is assigned at each year, according to different cutoffs for a positive result and different testing strategies (single test, or initial positive test requiring confirmation with a repeat test). Based on the test result and true LTBI status (incorporating this annual risk of TB infection) at each year, individuals in the population cycle between four Markov states: true negative, false negative, true positive, and false positive (Fig. 1B). All eligible (previously negative) individuals are assumed to undergo testing each year, and individuals who test positive (according to the selected cutoff and testing strategy) are assumed to be offered treatment for LTBI and therefore not subjected to further testing. QFT reversions are thus not explicitly modeled, as they have no bearing on outcome. Our primary outcome is the proportion of the population in each of the four states (with particular interest in the false-positive state) at the end of ten years. Key secondary outcomes include the number of QFT tests that would be performed over ten years and the proportion of people with true TB infection who would be properly identified as true positive in the first year after the infection occurred (i.e., those at greatest risk of progression to active disease and therefore greatest priority for preventive therapy, despite the relatively low accuracy of QFTs in predicting progression to active TB^15^).

### Sensitivity Analyses

We evaluated outcomes using the manufacturer’s recommended conversion cutoff for a positive test result (≥0.35 IU/mL), as well as higher cutoffs at regular intervals (≥0.5, ≥0.75, and ≥1.0 IU/mL). In addition, we modeled the correlation between initial and confirmatory tests in separate analyses as a Pearson correlation coefficient of 0.0 (presumed independent tests), 0.5, and 0.9 (best reflection of available data)^25^. We evaluated a wide range of thresholds for defining the three QFT classes (not shown), choosing the thresholds above based on natural breaks in the data distribution and a desire to be conservative in our assumptions. To evaluate the impact of the different modeled sources of variability on the outcomes of serial testing, we performed additional model runs in which each source of variability was removed from the model. Finally, we ran 1,000 separate model simulations (of 10,000 individuals each) to construct 95% uncertainty ranges (95% URs) as the 2.5^th^ and 97.5^th^ percentile of results across all simulations.

## Results

In the base case scenario (0.1% annual risk of TB infection), 0.9% (95%UR: 0.7–1.1%) of the population experienced a true incident TB infection within ten years. Under a testing strategy in which a QFT conversion was defined as an initial value of <0.35 IU/mL followed by a subsequent test ≥0.35 IU/mL–and with no confirmatory testing–24.6% (95%UR: 23.8–25.5%) of the population was projected to test false positive by the end of ten years, versus 0.85% (95% UR: 0.68–1.04%) true positives: a ratio of 29 false positives for every true positive TB infection diagnosed. Under this strategy, 23.5% (95%UR: 15.4 −33.7%) of patients with true incident infections were missed on the first test after infection. Including variability due to TST boosting nearly doubled the number of false positives, while removing variability due to incubation delays and blood volume (i.e., limiting variability to within-subject variability) did not materially change results (data not shown).

**Figure 2:**
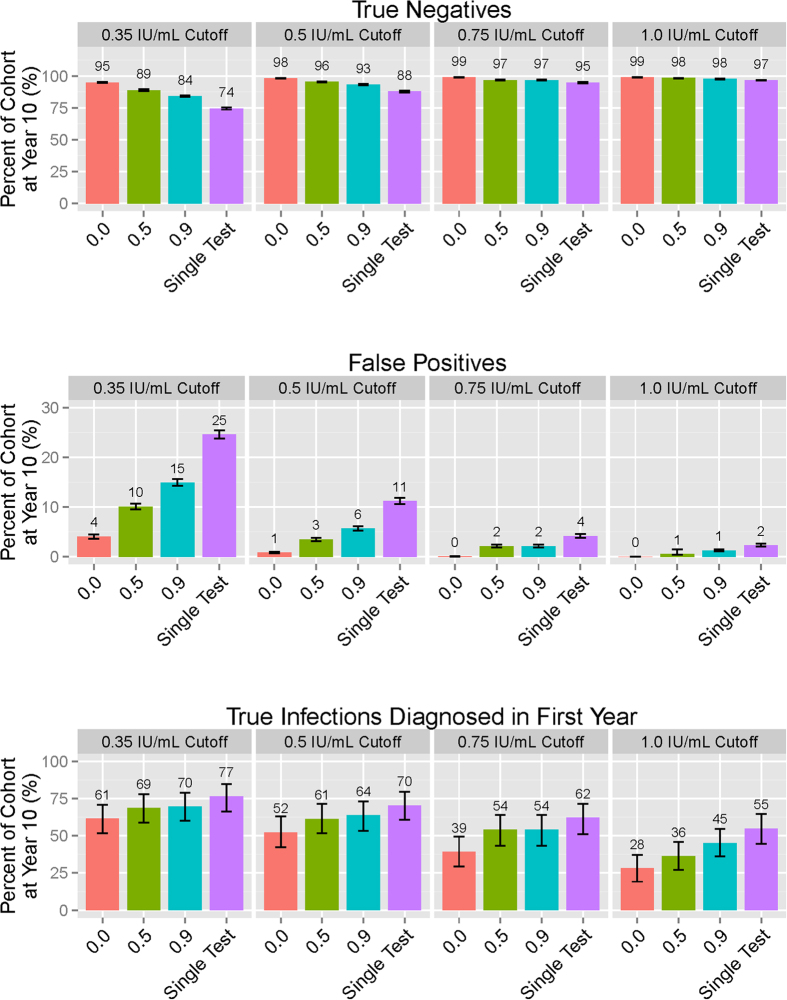
Cumulative Outcomes of Serial QuantiFERON-TB (QFT) Testing After Ten Years. The three panels respectively display proportions of the initial cohort classified as true negatives, false positives, and true infections diagnosed in the first year of infection at the end of ten years for each correlation-cutoff scenario. Correlations represent the Pearson correlation between initial test and confirmatory test (given if initial test was positive).

**Figure 3:**
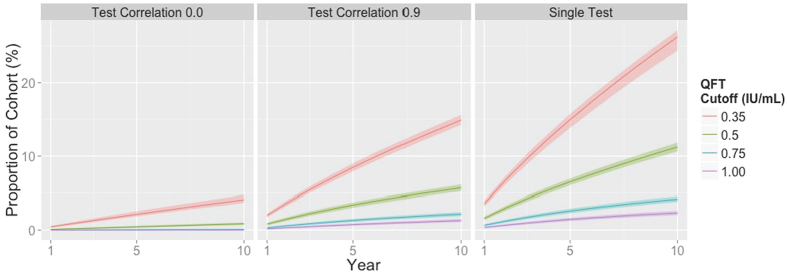
Proportion of a Hypothetical Population of Healthcare Workers Testing False-Positive for Tuberculosis Infection Using QuantiFERON-TB. Lines show the cumulative proportion of healthcare workers expected to test false-positive for latent tuberculosis infection (LTBI) if tested serially with QuantiFERON-TB on an annual basis, using different cutoffs for a positive test (shown in different colors) and different testing strategies (independent confirmatory test on the left, confirmatory test with Pearson correlation of 0.9 in the middle, and no confirmatory testing on the right).

We then evaluated different strategies for reducing the false positive: true positive ratio, by instituting a higher cutoff for conversion and/or requiring confirmation of positive tests, under different assumptions about the correlation between the initial and confirmatory test (Fig. 2). Increasing the cutoff for a conversion to 1.0 IU/mL reduced the proportion of the population testing false-positive in ten years to 2.3% (95%UR: 2.0–2.6%) (Figs 2 and 3), but at the expense of reducing the percentage of true TB infections diagnosed within the first year after infection to 54.8% (95%UR: 44.6–64.5%).

The value of a confirmatory test depended strongly on the modeled correlation between initial and confirmatory test results. If the hypothetical correlation between initial and confirmatory test was high, outcomes were very similar whether or not confirmatory testing was performed. (See Fig. 2, “0.9” bars, and Fig. 3, middle and right-hand panels.) By contrast, if confirmatory tests were uncorrelated with the initial test result (Fig. 2, “0.0” bars, and Fig. 3, left panel), confirmatory testing even at a threshold of 0.35 IU/mL reduced the ten-year false positive percentage from 24.6% to 4.1% (95%UR: 3.7–4.5%). In this case, confirmatory testing also reduced the proportion of true infections detected within one year from 76.5% to 61.5% (95%UR: 51.6–70.9%).

At a higher annual risk of TB infection, the proportions of true positive tests increased, but the number of false positive tests remained the same, such that the false positive: true positive ratio declined (Table 2). In an extreme scenario of 1.0% annual risk of TB infection per year (1 of every 100 individuals tested actually has incident TB infection), assuming a correlation between initial and confirmatory testing of 0.5, a cutoff of 1.0 IU/mL, and only within-subject variability, the number of individuals testing true positive (7.95%) was much greater than the number testing false positive (0.55%). As a result, the post-test probability of a positive test representing true TB infection in this scenario was 0.94, versus 0.035 in the base case (0.1% annual risk of infection, no confirmatory testing, cutoff of 0.35 IU/mL).

## Discussion

Using QFT for annual serial testing among HCWs in a low-incidence setting such as North America may lead to tremendous over-diagnosis of QFT conversions, and likely over-treatment of patients testing false-positive for LTBI. Using the manufacturer’s recommended diagnostic cutoff as the cutoff for conversion, and without a confirmatory test, resulted in 24.6% of HCWs being diagnosed with false positive conversions over 10 years of serial testing. If this number of individuals were unnecessarily treated, adverse events and costs associated with treatment, monitoring, and side effects would be substantial, given the millions of HCWs who undergo serial testing in North America. Based on the present analysis, serial testing in the absence of confirmatory tests or alternative conversion cutoffs should therefore be discouraged in low-incidence settings.

Using a higher cutoff for a QFT conversion, or confirming positive results with a second test (assuming low correlation between those tests) can substantially reduce the number of false-positive results, but at the consequence of diagnosing fewer people within the first year after true infection. Furthermore, in settings with a low annual risk of TB infection (1 per 1,000 per year or less), the positive predictive value of serial QFT testing, even with high cutoffs and independent confirmatory testing, can be 60% or lower. For example, if preventive therapy costs $430 per treatment course^26^, and assuming that preventive therapy is taken as prescribed, a serial QFT strategy using a single annual test with a cutoff of 0.35 IU/mL (29 false-positives for every true-positive LTBI case) could cost $13,000 per true-positive LTBI case treated, in treatment costs alone–a figure comparable to the cost of treating a case of active TB^27^. In a population of 10,000 individuals, if all (2350) individuals testing false-positive under this strategy were to take preventive therapy, 87–115 people (3.7–4.9%) would be expected to experience an adverse event sufficient to result in permanent drug discontinuation^28^. Using a confirmatory test with correlation of 0.5 and a cutoff of 0.35 IU/mL could reduce the cost per true-positive LTBI case treated to $5,400, but at the expense of approximately two more cases of TB infection going undiagnosed (double the false negative rate of an annual single QFT serial screening strategy). Decisions of whether to adopt such higher cutoffs in the context of serial testing must therefore consider the underlying risk of TB infection, weighing the benefits of fewer false-positive tests against the potential harm of failing to promptly diagnose individuals with true TB infection.

Confirmatory testing has been discussed before^29^ but, to date, no guidance exists regarding its appropriate implementation. Data to describe the true correlation between initial and confirmatory QFT test results and estimates of cost-effectiveness could be helpful in this regard. Despite this uncertainty, many hospitals have implemented confirmatory testing as a method to reduce false positive results and avoid unnecessary treatment^30^,^31^. Our model demonstrates the wide range of clinical outcomes possible, depending on the true correlation of results between initial and confirmatory testing. Given that confirmatory QFT testing is widely employed, more accurately characterizing this correlation should be a high priority for future research.

As with any modeling study, we are limited by the accuracy and appropriateness of the model and parameter assumptions employed. While we performed sensitivity analyses around the sources of variability, distributions employed, cutoffs for the underlying QFT categories and other parameters, we cannot exclude the possibility of having not modeled or not modeled accurately a particular unknown source of variability. In the assignment of initial QFT values and the distribution of within-subject variability, our analysis relied heavily on data from the largest study of IGRAs in North American HCWs [8]; however, this study collated QFT measurements from laboratories employing potentially diverse methodologies. Such pooling of data from different settings may increase uncertainty in the data underlying our results. In choosing categories and cutoffs we employed conservative estimates likely to be favorable to QFT; to the extent that we have underestimated the probability of QFT conversions under each source of variability, our results may underestimate the number of false-positive results that could be expected under each serial QFT testing strategy. Furthermore, as the focus of this modeling work was on serial QFT testing, we did not consider serial TST or T-SPOT.TB algorithms; further work comparing serial QFT to serial TST or T-SPOT.TB testing–incorporating year-to-year variability in each test–could add further value.

This model demonstrates the potential pitfalls of blindly using cutoffs designed for single tests in the setting of serial testing, particularly in settings of low TB incidence (i.e., low positive predictive value of QFT conversions). Simulation can help us understand these consequences in the absence of large prospective cohort studies. In realistic settings of low risk of TB infection, even with high cutoffs and confirmation, the post-test probability of a positive QFT test on serial testing may still only approach 60%, while annual testing with a single QFT using the recommended diagnostic cutoff may cause over 25% of individuals to have a false-positive test over a span of ten years. The value of serial testing of HCWs should therefore be discouraged in the North American setting, where TB incidence is now at an all-time low.

## Additional Information

**How to cite this article**: Moses, M. W. *et al.* Serial testing for latent tuberculosis using QuantiFERON-TB Gold In-Tube: A Markov model. *Sci. Rep.* **6**, 30781; doi: 10.1038/srep30781 (2016).

## Figures and Tables

**Table 1:** Parameter Distributions for a Markov Model of Serial QFT Testing.

Parameter	Value*	Distribution	Source
Distributions Used to Account for Within-Subject Variability:			
Initial QFT (to determine QFT class)	0.00 (0.05)	Gamma	8
Annual “Underlying” QFT Result			8,10,18
QFT class 0–0.19 IU/mL	0.00 (0.11)	Gamma	
QFT class 0.20 IU/mL–0.99 IU/mL	0.00 (0.61)	Gamma	
QFT class ≥1.00 IU/mL	10 (2.82)	Gamma	
Additional Sources of Variability:			
Probability of Event			Assumed
6-Hour Pre-incubation Delay	0.05	Binomial	
12-Hour Pre-incubation Delay	0.05	Binomial	
0.8 mL Blood Sample	0.05	Binomial	
1.2 mL Blood Sample	0.05	Binomial	
Annual Risk of TB Infection	0.01	Binomial	
Change in QFT Result According To:			
6 Hour Pre-analytical Delay			20,21
QFT class 0–0.19 IU/mL	0.02 [−0.14, 4.23]	Beta	
QFT class 0.20 IU/mL–0.99 IU/mL	−0.16 [−0.60, 0.19]	Beta	
QFT class ≥1.00 IU/mL	−2.03 [−7.97, 0.86]	Beta	
12 Hour Pre-analytical Delay			20,21
QFT class 0–0.19 IU/mL	0.01 [−0.13, 0.61]	Beta	
QFT class 0.20 IU/mL–0.99 IU/mL	−0.16 [−0.89, 0.06]	Beta	
QFT class ≥1.00 IU/mL	−2.85 [−7.16, 0.00]	Beta	
0.8 mL Blood Volume			19
QFT class 0–0.19 IU/mL	0.03 [−0.02, 0.33]	Beta	
QFT class 0.20 IU/mL–0.99 IU/mL	0.35 [−0.11, 1.36]	Beta	
QFT class ≥1.00 IU/mL	2.68 [−1.11, 7.32]	Beta	
1.2 mL Blood Volume			19
QFT class 0–0.19 IU/mL	0.01 [−0.11, 0.59]	Beta	
QFT class 0.20 IU/mL–0.99 IU/mL	−0.28 [−0.42, −0.05]	Beta	
QFT class ≥1.00 IU/mL	−0.97 [−2.13, 0.09]	Beta	
TST Boosting (excluded from base case)			8,22, 23, 24
QFT class 0–0.19 IU/mL	0.04 [−0.16, 7.46]	Beta	
QFT class 0.20 IU/mL–0.99 IU/mL	1.13 [−0.85, 9.22]	Beta	
QFT class ≥1.00 IU/mL	2.86 [−1.56, 8.59]	Beta	

^*^Gamma distributions show mode (standard deviation), each rounded to the nearest 0.01. Binomial distributions show probabilities. Beta distributions show mode (minimum, maximum), assuming an alpha (shape) value of 4. QFT: Quantiferon, TST: Tuberculin skin test, TB: tuberculosis.

**Table 2:** Outcomes of Serial QFT Testing Assuming Only Within-Subject Variability.

Annual Risk of TB infection (%)	0.10%	0.50%	1.00%
Outcomes at 10 Years (%)			
True Negative	98.4 (98.2, 98.7)	94.6 (94.1, 95.0)	89.9 (89.3, 90.5)
False Negative	0.17 (0.09, 0.25)	0.82 (0.64, 0.98)	1.57 (1.35, 1.83)
True Positive	0.82 (0.66, 1.01)	4.04 (3.66, 4.45)	7.95 (7.45, 8.49)
False Positive	0.57 (0.44, 0.73)	0.56 (0.42, 0.72)	0.55 (0.4, 0.7)
Number of QFT Tests Performed*	99,800 (99,800, 99,900)	99,100 (98,900, 99,300)	98,100 (97,900, 98,300)
TB Infections Diagnosed in the First Year	36.8 (27.3, 45.6)	36.7 (32.3, 41.3)	36.7 (33.7, 39.8)

Assuming a cutoff for conversion of 1.0 IU/mL and a confirmatory test with a Pearson correlation of 0.5. *Tests required to evaluate 10,000 individuals, followed over 10 years.
